# QTL analysis reveals genomic variants linked to high-temperature fermentation performance in the industrial yeast

**DOI:** 10.1186/s13068-019-1398-7

**Published:** 2019-03-19

**Authors:** Zhen Wang, Qi Qi, Yuping Lin, Yufeng Guo, Yanfang Liu, Qinhong Wang

**Affiliations:** 1 CAS Key Laboratory of Systems Microbial Biotechnology, Tianjin Institute of Industrial Biotechnology, Chinese Academy of Sciences, Tianjin, 300308 China; 2 University of Chinese Academy of Sciences, Beijing, 100049 China

**Keywords:** High-temperature fermentation (HTF), Pooled-segregant whole-genome sequence analysis, QTL mapping, Reciprocal hemizygosity analysis, Allele replacement, *Saccharomyces cerevisiae*

## Abstract

**Background:**

High-temperature fermentation is desirable for the industrial production of ethanol, which requires thermotolerant yeast strains. However, yeast thermotolerance is a complicated quantitative trait. The understanding of genetic basis behind high-temperature fermentation performance is still limited. Quantitative trait locus (QTL) mapping by pooled-segregant whole genome sequencing has been proved to be a powerful and reliable approach to identify the loci, genes and single nucleotide polymorphism (SNP) variants linked to quantitative traits of yeast.

**Results:**

One superior thermotolerant industrial strain and one inferior thermosensitive natural strain with distinct high-temperature fermentation performances were screened from 124 *Saccharomyces cerevisiae* strains as parent strains for crossing and segregant isolation. Based on QTL mapping by pooled-segregant whole genome sequencing as well as the subsequent reciprocal hemizygosity analysis (RHA) and allele replacement analysis, we identified and validated total eight causative genes in four QTLs that linked to high-temperature fermentation of yeast. Interestingly, loss of heterozygosity in five of the eight causative genes including *RXT2*, *ECM24*, *CSC1*, *IRA2* and *AVO1* exhibited positive effects on high-temperature fermentation. Principal component analysis (PCA) of high-temperature fermentation data from all the RHA and allele replacement strains of those eight genes distinguished three superior parent alleles including *VPS34*, *VID24* and *DAP1* to be greatly beneficial to high-temperature fermentation in contrast to their inferior parent alleles. Strikingly, physiological impacts of the superior parent alleles of *VPS34*, *VID24* and *DAP1* converged on cell membrane by increasing trehalose accumulation or reducing membrane fluidity.

**Conclusions:**

This work revealed eight novel causative genes and SNP variants closely associated with high-temperature fermentation performance. Among these genes, *VPS34* and *DAP1* would be good targets for improving high-temperature fermentation of the industrial yeast. It also showed that loss of heterozygosity of causative genes could contribute to the improvement of high-temperature fermentation capacities. Our findings would provide guides to develop more robust and thermotolerant strains for the industrial production of ethanol.

**Electronic supplementary material:**

The online version of this article (10.1186/s13068-019-1398-7) contains supplementary material, which is available to authorized users.

## Background

*Saccharomyces cerevisiae* has been widely used for the production of various fuels and chemicals, more recently, eco-friendly bioethanol [1, 2]. Although robust industrial *S. cerevisiae* strains produce ethanol from agricultural wastes with high yield and productivity, the urgent demand of larger production and minimum costs is still challenging. Improved thermotolerance performance can address this obstacle to some extent, since high-temperature fermentation can greatly reduce cooling costs, increase cell growth, viability and ethanol productivity via facilitating the synchronization of saccharification and fermentation [3, 4]. However, thermotolerance is a complex quantitative trait and determined by a complicated mechanism referring to the interaction of many genes [5]. Thus, it is very challenging to develop robust *S. cerevisiae* strains with enhanced thermotolerance to meet industrial requirement.

Many efforts have been made to understand the molecular mechanisms and genetic determinants underlying yeast thermotolerance, but most of them focused on laboratory strains, which display much lower thermal tolerance than the robust industrial and natural yeast strains [6]. Previous study indicated that industrial yeast has evolved complex but subtle mechanisms to protect the organism from high-temperature lesion by activating and regulating of specific thermal tolerance-related genes to synthesize specific compounds [7]. To identify novel genes and elucidate the intricate mechanism of thermotolerance, many methods were developed [8–12]. Although these approaches have disclosed a number of causative genes and revealed some compounds, e.g. sterol composition, for responding to the thermal stress, identification of quantitative trait genes still faced with tremendous challenges, including variable contributions of quantitative trait loci (QTL), epistasis [13], genetic heterogeneity [14], etc.

With the rapidly development of high-throughput genome sequencing, pooled-segregant whole genome sequencing technology has been developed for efficiently mapping QTLs related to complex traits [15, 16]. Subsequent genetic approaches, such as reciprocal hemizygosity analysis (RHA) and allele replacement analysis, accelerated identification of the causative genes linked to superior phenotypes [17]. *S. cerevisiae* as a model organism is renowned for the acquisition of abundant genetic markers [18], the ease of introduction of precise genetic modification and the convenience of performing experimental crosses [19], thus perfectly suitable for the application of QTL methodology to disclose complex traits. The efficient methodology has facilitated identification of several genomic regions and causative genes related to the complex traits in *S. cerevisiae*, including thermotolerance, ethanol tolerance, glycerol yield, etc. [5, 20–22]. However, up to now, the underlying molecular mechanisms of thermotolerance in *S. cerevisiae* are still unclear, and the identification of novel causative genes continues to be of interest to accelerate the breeding of robust yeast strains with improved high-temperature fermentation performance.

**Fig. 1:**
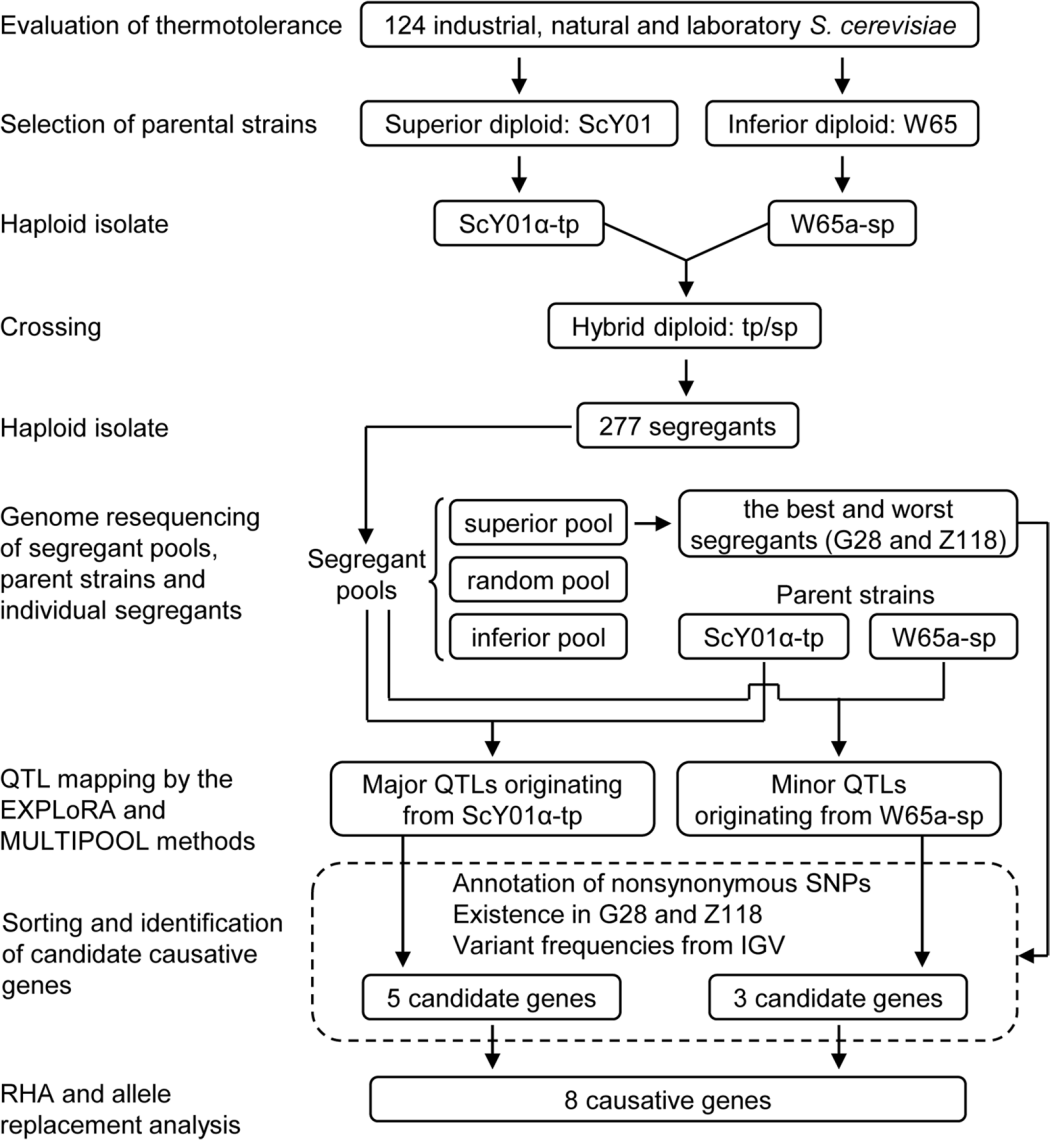
Scheme of identification of causative genes linked to the thermotolerance phenotype. One superior thermotolerant strain ScY01 and one inferior thermosensitive strain W65 were selected from 124 *S. cerevisiae* strains based on evaluation of thermotolerance. The haploid segregants of two parent strains with *HO* gene deletion were generated and crossed to create the hybrid diploid strain tp/sp. Total 277 segregants were sporulated from the hybrid strain and selected for the superior, random and inferior pools based on evaluation of thermotolerance. Genomic DNA was extracted from these three pools as well as two parent strains and the best and worst spores in the superior pool and subjected to genome resequencing. QTL mapping analyses were performed using the EXPloRA and MULTIPOOL methods. To identify candidate causative genes, the SNPs in QTLs were annotated, and the nonsynonymous SNPs in coding regions were sorted out according to their existences in G28 and Z118 and their manually checked frequencies using Integrative Genomics Viewer (IGV). Two major QTLs and two minor QTLs were identified to originate from the superior and inferior parent strain, in which five and three genes contained nonsynonymous single nucleotide polymorphism (SNP) variants, respectively. Reciprocal hemizygosity analysis (RHA) and allele replacement analysis further revealed two causative genes in major QTLs and one causative gene in minor QTLs

In this study, to uncover genetic determinants linked to high-temperature fermentation performance of the industrial yeast, QTL mapping by pooled-segregant whole genome sequence analysis and subsequent validation by RHA and allele replacement analysis were performed. The scheme of this work was shown in Fig. 1. Total eight genes containing nonsynonymous SNP variants in two major QTLs linked to the superior parent and two minor QTLs linked to the inferior parent were identified and validated to be causative genes tightly associated with thermotolerance. Among these genes, loss of heterozygosity in *RXT2*, *ECM24*, *CSC1*, *IRA2* and *AVO1* seemed to play beneficial roles in developing thermotolerance; meanwhile, the superior parent alleles of *VPS34*, *VID24* and *DAP1* were distinguished to be greatly beneficial to high-temperature fermentation in contrast to their inferior parent alleles, due to their positive effects on improving protective function of cell membrane against thermal stress. This study improved our understanding of genetic basis behind thermotolerance, and identified more new causative genes linked to yeast thermotolerance, thus providing more guidance to enhance thermotolerance of industrial yeast strains.

## Results

### Selection of parent strains for genetic mapping of thermotolerance

**Fig. 2:**
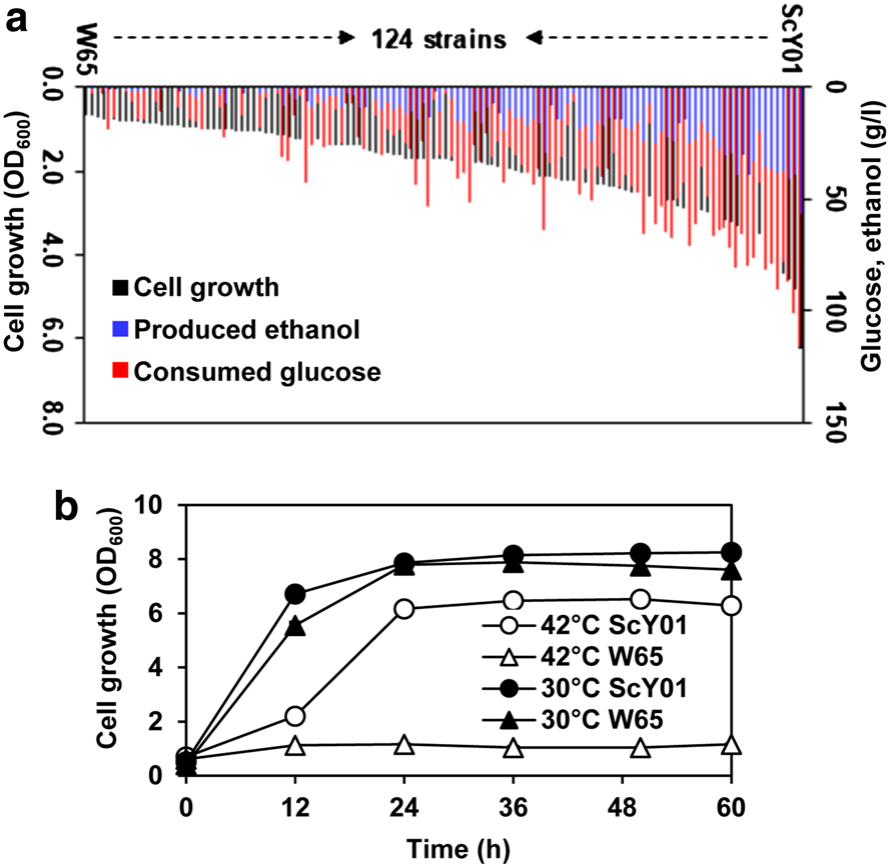
Thermotolerance of 124 *S. cerevisiae* strains including two parent strains ScY01 and W65. **a** Cell growth (black bar), consumed glucose (red bar) and produced ethanol (blue bar) at 42 °C for 36 h. Cells were grown in YP medium containing 200 g/l glucose. **b** Cell growth of two parent strains ScY01 and W65 at 42 °C and 30 °C. Cells were grown in 100 ml Erlenmeyer flasks containing 50 ml YP medium with 200 g/l glucose. Data represent the mean and standard error of duplicate cultures at each condition (error bars are covered by symbols). Initial OD_600_ of 0.5 was used for all the fermentations. In panel **b**, data represent the mean and standard error of duplicate cultures at each condition

Total 124 natural, laboratory and industrial isolates of *S. cerevisiae* collected in our lab (Additional file 1: Table S1) were evaluated for their high-temperature fermentation performances. The OD_600_ values representing cell growth at 42 °C for 36 h ranged from 0.66 to 6.24 (Fig. 2a, Additional file 2: Table S2), showing that the strain ScY01 had the highest cell growth while W65 had the lowest cell growth under thermal stress conditions. Meanwhile, ScY01 consumed the highest amount of glucose (116.0 g/l) and produced the highest amount of ethanol (57.3 g/l) at 42 °C (Fig. 2a, Additional file 2: Table S2). By contrast, W65 almost had no glucose consumption and ethanol production at 42 °C. ScY01 derived from the industrial strain Ethanol Red through adaptive evolution at high temperature [11], whereas W65 is a natural isolate. Cell growth profiles at 42 °C and 30 °C further confirmed that ScY01 was significantly more thermotolerant than W65 at elevated temperature (Fig. 2b), while both strains had no significant differences of cell growth at normal temperature. Therefore, ScY01 and W65 were chosen as the superior and inferior strains for genetic mapping of thermotolerance, respectively.

Both ScY01 and W65 were separately sporulated to generate the MATα and MATa haploid segregants, named ScY01α and W65a (Additional file 1: Table S1), respectively. To obtain stable haploids for genetic mapping, the *HO* gene in ScY01α and W65a were further knocked out by inserting zeocin- or geneticin-resistance cassettes. The resulting haploid parent strains were named ScY01α-tp and W65a-sp, respectively.

### Screening of the superior, inferior and random pools of segregants for genome sequencing

The parent haploid strains ScY01α-tp and W65a-sp were crossed to obtain the hybrid diploid strain tp × sp and then sporulated. Since ScY01α-tp and W65a-sp had zeocin- or geneticin-resistance cassettes at *HO* locus, successfully segregated haploid spores should only inherit one drug resistance capacity of either zeocin or geneticin. Combining with the subsequent diagnostic PCR for the MAT locus, we isolated 107 haploid segregants on geneticin selective plates and 170 haploid segregants on zeocin selective plates. Total 277 haploid segregants were isolated and tested for their thermotolerance capacities to screen the ten most thermotolerant or thermosensitive segregants for the superior pool and the inferior pool, respectively, as well as ten random segregants for the random pool for genome sequencing.

**Fig. 3:**
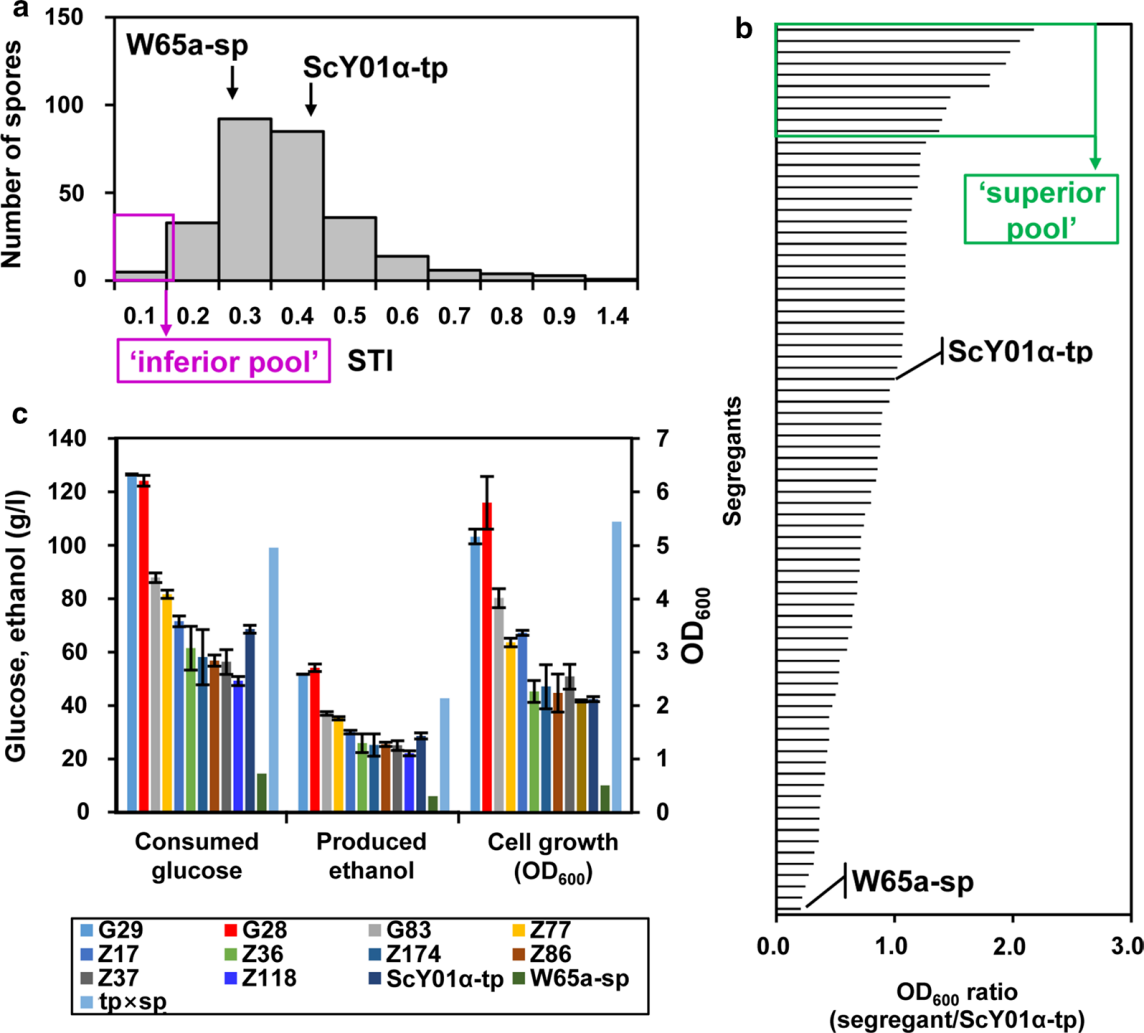
Selection of superior, inferior and random pools for genome sequencing by evaluating thermotolerance capacities of segregants. **a** The distribution of the STI values in 277 haploid segregants from the hybrid of the two parent haploid strains ScY01α-tp and W65a-sp. Seventy-seven segregants showing higher STI values (> 0.38) than the superior parent ScY01α-tp were selected as superior segregants. Forty-nine segregants showing lower STI values (< 0.22) than the inferior parent W65a-sp were selected as inferior segregants. Ten segregants showing the ten lowest STI values (0.09 to 0.11) were selected as the most thermosensitive segregants and assembled in the inferior pool. Cell growth experiments were carried out in triplicates for each strain in 96-well plates with 1 ml YPD medium at 42 °C and 30 °C. **b** Cell growth comparison of the 77 segregants and W65a-sp with ScY01α-tp at 42 °C. Thirty-one segregants showed higher cell growth than ScY01α-tp at 42 °C. Ten segregants showing the ten highest OD_600_ ratios (1.37 to 2.17) than ScY01α-tp were selected as the most thermosensitive segregants and assembled in the superior pool. **c** Fermentation capacities of ten segregants in the superior pool at 42 °C. Fermentation experiments were conducted in 100 ml Erlenmeyer flasks containing 50 ml YP medium with 200 g/l glucose at 42 °C. Consumed glucose, produced ethanol and cell growth were measured after incubation for 36 h. Data represent the mean and standard error of duplicate cultures at each condition. Excluding the segregants in superior and inferior pools, ten of the rest segregants were selected and assembled in the random pool

The distribution of the stress tolerance index (STI) values (calculated as the ratio of the OD_600_ at 42 °C versus the OD_600_ at 30 °C measured at the 16-h time point) in 277 haploid segregants is shown in Fig. 3a. Apparent continuous variation as well as normal frequency distribution of STI in the haploid segregants from the hybrid tp × sp indicated yeast thermotolerance as a quantitative trait. Among them, 49 segregants showed lower STI values (< 0.22) than the inferior parent W65a-sp, while 77 segregants showed higher STI values (> 0.38) than the superior parent ScY01α-tp. Thus, ten segregants showing the 10 lowest STI values (0.09 to 0.11) were selected as the most thermosensitive segregants and assembled in the inferior pool (Fig. 3a). To further narrow down superior segregants, cell growths of those 77 segregants at 42 °C were compared with ScY01α-tp (Fig. 3b). Among them, 31 segregants showed higher cell growth than ScY01α-tp at 42 °C. Thus, ten segregants showing the ten highest OD_600_ ratios (1.37 to 2.17) than ScY01α-tp were selected as the most thermosensitive segregants and assembled in the superior pool. Finally, excluding the segregants in superior and inferior pools, ten of the rest segregants were randomly selected and assembled in the random pool.

Additionally, fermentation capacities of the ten segregants in the superior pool as well as parent strains were evaluated (Fig. 3c). After 36 h incubation at 42 °C, the thermotolerant parent strain ScY01α-tp consumed 68.6 ± 1.5 g/l glucose, produced 28.6 ± 1.1 g/l ethanol and resulted in cell growth of 4.12 ± 0.04 OD_600_. By contrast, the thermosensitive parent strain W65a-sp, which consumed 14.4 ± 0.3 g/l glucose, produced 6.0 ± 0.2 g/l ethanol, and resulted in cell growth of 0.50 ± 0.01 OD_600_, showing much lower fermentation capacity in contrast to ScY01α-tp. The hybrid strain tp × sp exhibited higher fermentation capacity than both the haploid parent strains, which might be partially due to ploidy-driven adaptation in cell physiology as previously reported [23]. Remarkably, two segregants G29 and G28 showed higher capacities of glucose consumption and ethanol accumulation than the hybrid strain tp × sp and the superior parent ScY01α-tp, implicating unknown genetic factors beyond the impacts of ploidy and the superior parent on cell physiology. In addition, G28 showed slightly higher ethanol accumulation than G29. On the other hand, the segregant Z118 showed the worst fermentation capacity. Thus, to facilitate QTL mapping based on pooled-segregant whole-genome sequence analysis, the best and the worst spores (G28 and Z118) from the superior pool were also selected for genome sequencing.

### Identification of QTLs and candidate causative genes by pooled-segregant whole-genome sequence analysis

**Fig. 4:**
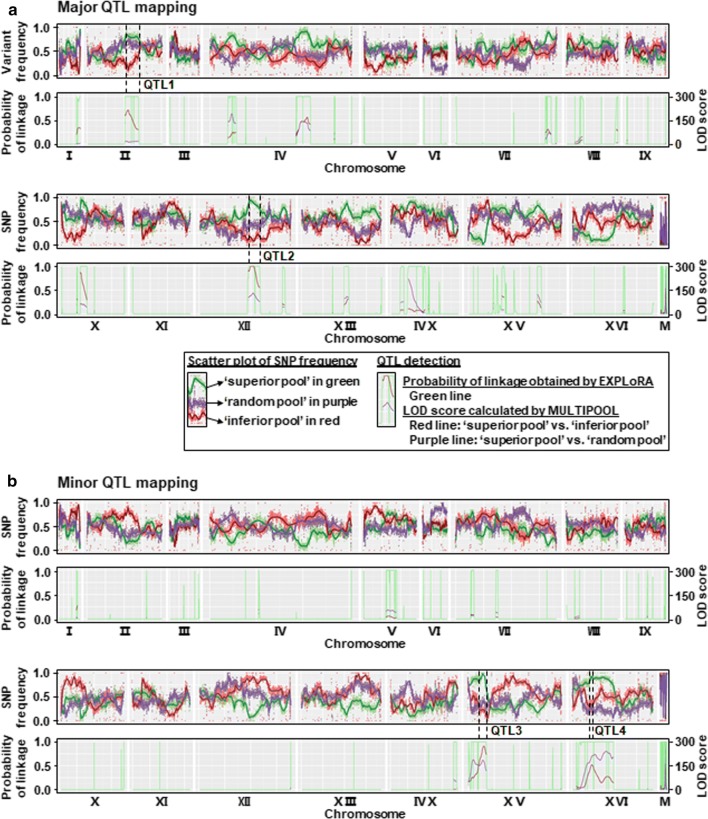
Mapping of major (**a**) and minor (**b**) thermotolerance-related QTLs by pooled-segregant whole-genome sequence analysis. **a** In major QTL mapping, the SNP frequencies refer to the percentage of the SNP nucleotide in the pools originating from the thermotolerant parent strain ScY01α-tp. **b** In minor QTL mapping, the SNP frequencies refer to the percentage of the SNP nucleotide in the pools originating from the thermosensitive parent strain W65a-tp. In the upper panels of **a** and **b**, scatter plots of SNP frequency versus chromosome are shown. The raw data of SNP frequencies are shown as dots, smoothened using a Linear Mixed Model [30] and shown in bold lines. Green, red and purple dots and lines represent the raw data and smoothed data of SNP frequencies in superior pool, inferior pool and random pool, respectively. In the bottom panels of **a** and **b**, QTL detections using the EXPLoRA and MULTIPPOL methods are shown. The green line represents the probability of linkage obtained by EXPLoRA, where peak regions showed higher SNP frequencies than 0.5 and were, therefore, detected as QTLs. The red line represents LOD scores in superior pool versus inferior pool calculated by MULTIPOOL, whereas the purple line represents LOD scores in superior pool versus random pool. When both maximum LOD scores were higher than 5, this locus was detected as a QTL by MULTIPOOL. QTLs were further narrowed down by analysing whether nonsynonymous amino acid changes were present. Eventually, two major QTLs named QTL1 and QTL2 as well as two minor QTLs named QTL3 and QTL4 were identified

To identify the genetic basis underlying yeast thermotolerance, seven samples, which were two parent strains ScY01α-tp and W65a-sp showing distinct thermotolerance capacities, three segregant pools including the superior, inferior and random pools derived from these two parents, and two individual segregants that were the best and worst segregants G28 and Z118 in the superior pool, were subjected to whole-genome sequencing for QTL mapping analysis. Single nucleotide polymorphisms (SNPs) in these seven samples were separately extracted from their genomic alignments with the sequence of the reference S288c genome. Total 35,459 quality-filtered and discordant SNPs from two parent strains were used as genetic makers (Additional file 3: Dataset S1). Usually, thermotolerance-related SNP variants in the superior pool are expected to dominantly inherit from the superior parent. However, previous studies have demonstrated the presence of recessive mutations linked to yeast stress tolerance in the inferior parent [5, 24], suggesting that the inferior parent could also pass thermotolerance-related SNP variants on to segregants in the superior pool. Therefore, we detected the major QTLs originating from the superior parent and the minor QTLs inherited from the inferior parents, respectively. Correspondingly, the SNP variant frequencies in the three segregant pools were calculated as the percentages of the SNP nucleotides originating from the superior or inferior parent for mapping major or minor QTLs. The raw SNP frequencies were plotted against the chromosomal position and smoothened by using a Linear Mixed Model [25] (Fig. 4, upper panel). Linkage analysis of QTLs was further performed using the EXPLoRA and MULTIPOOL methods [26] (Fig. 4, bottom panel). Overall, the numbers of QTLs identified by these two methods were similar (Table 1; Additional file 4: Dataset 2; Additional file 5: Dataset 3). However, the average lengths of major and minor QTLs identified by EXPLoRA were 47-kb or 69-kb, which were refined to 20-kb or 12-kb by MULTIPOOL (Table 1). Meanwhile, the numbers of nonsynonymous variants and affected genes were narrowed down.

**Table 1:** QTL mapping by EXPLoRA and MULTIPOOL methods

	Method	Number of QTL	Average length (kb)	Number of nonsynonymous variants	Number of affected genes
Major QTL	EXPLoRA	24	47	744	297
	MULTIPOOL	22	20	292	119
Minor QTL	EXPLoRA	13	69	521	233
	MULTIPOOL	11	12	86	40

To subtly identify candidate causative genes linked to thermotolerance, all the SNP variants in the QTLs identified by MULTIPOOL were localized to coding and non-coding regions and annotated to be synonymous and nonsynonymous (Additional file 5: Dataset S3). Furthermore, the nonsynonymous SNPs in coding regions, which in two sequenced individual spores G28 and Z118 were similar to those in the parent strains and also consisted with those in the superior pool, were sorted out and manually checked for their frequencies using Integrative Genomics Viewer (IGV) [27–29]. We estimated that the SNP frequencies (count of SNP-containing reads/total count of mapped reads) in QTLs linked to thermotolerance could be high in the superior pool, but low in the inferior pool, and simultaneously at the median value of around 0.5. Only the variants in major QTLs meeting the criteria of allele frequencies with ≤ 10% in the inferior pool, ≥ 75% in the superior pool and around 50% in the random pool as well as the variants in minor QTLs meeting the criteria of allele frequencies with ≤ 25% in the inferior pool, ≥ 75% in the superior pool and around 50% in the random pool were considered to be causative variant candidates related to thermotolerance (Additional file 5: Dataset S3). Therefore, the genes affected by these causative variants were considered as candidate causative genes, and the QTLs containing these candidate causative genes were fine-mapped (Table 2). In total, two major QTLs, QTL1 and QTL2, were localized on chromosome II and XII (Fig. 4a) and contained two (*RXT2* and *VID24*) and three affected genes (*ECM22*, *VPS34* and *CSC1*) by nonsynonymous causative variant candidates. Two minor QTLs, QTL3 and QTL4, were localized on chromosome XV and XVI (Fig. 4b) and contained two (*IRA2* and *AVO1*) and one (*DAP1*) affected genes by nonsynonymous causative variant candidates (Table 2). Total eight candidate causative genes were identified by pooled-segregant whole-genome sequence analysis.

**Table 2:** Genes with nonsynonymous variants in two major and two minor QTLs

QTLs	Chr	Start (bp)	End (bp)	Length (bp)	LOD score	Affected gene	Mutation (S288c genome as a reference)	
							ScY01α-tp	W65a-sp
Major QTLs								
QTL1	II	408,800	553,700	144,900	17	*RXT2*	332 G>C (R111G)	Wild type
						*VID24*	154 C>T (P52S)	Wild type
QTL2	XII	595,800	633,500	37,700	300	*ECM22*	1954 G>A (G652S)	Wild type
						*VPS34*	1773 C>G (D591E)	Wild type
						*CSC1*	1126 C>A (Q376K)	Wild type
Minor QTLs								
QTL3	XV	174,500	184,900	10,400	272	*IRA2*	Wild type	7222 C>A (P2408T)
						*AVO1*	Wild type	2558 T>C (V853A)
QTL4	XVI	228,200	238,100	9900	155	*DAP1*	Wild type	115 G>A (V39I)

### Validation of causative genes in the QTLs

**Fig. 5:**
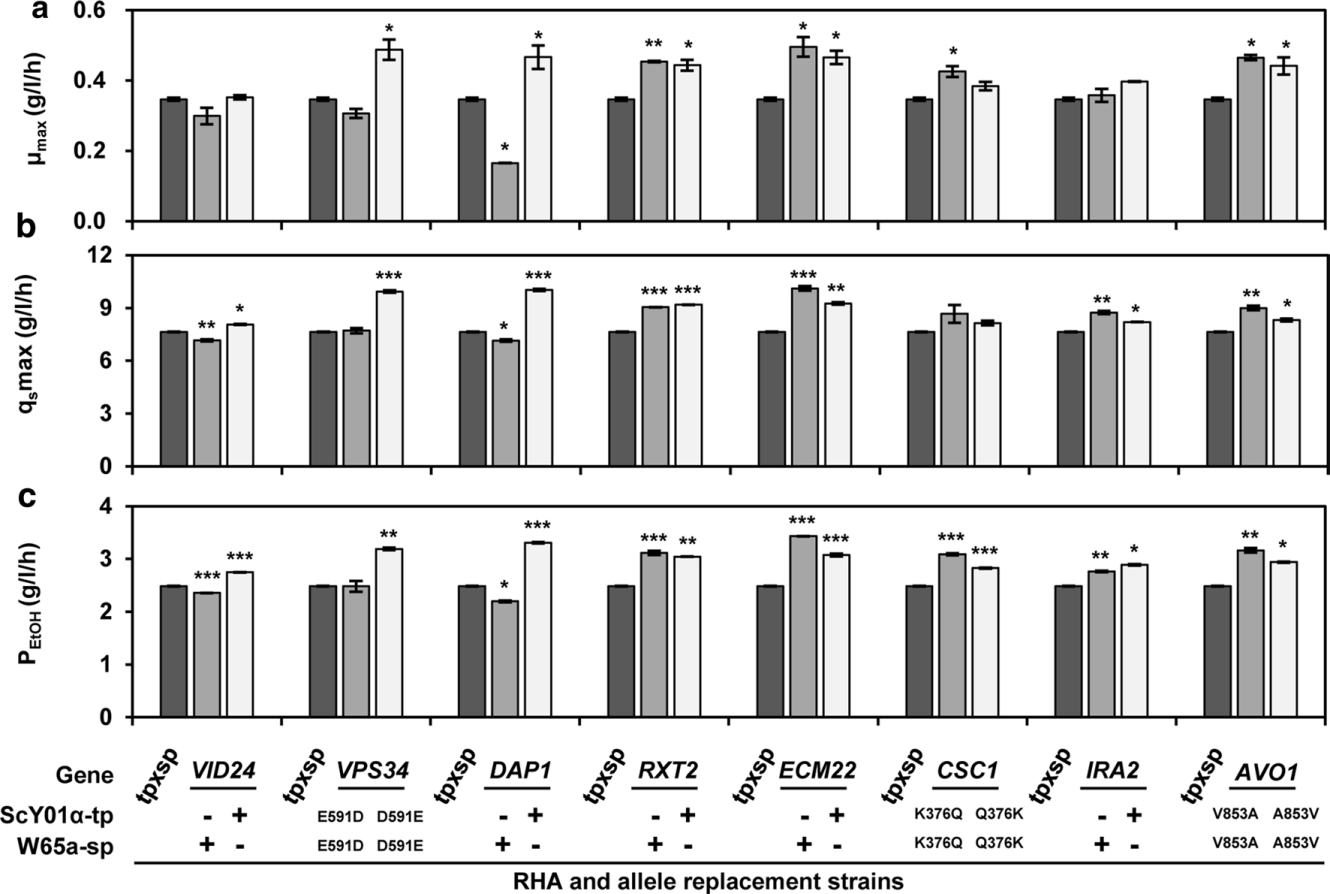
Identification of the causative genes using RHA and allele replacement methods. **a** Maximum cell growth rate (*μ*_max_). **b** Glucose-consumption rate (*q*_s_max). **c** Ethanol productivity (*P*_EtOH_). The RHA and allele replacement strains are detailed in Additional file 1: Table S1. High-temperature fermentation capacities were evaluated at 42 °C in 100 ml Erlenmeyer flasks containing 50 ml YP medium with 200 g/l glucose at 220 rpm. Data represent the mean and standard error of duplicate cultures at each condition. Statistical analysis for each group of three strains including the control strain tp × sp and two hemizygotes or homozygotes of each gene was performed using one-way ANOVA followed by Tukey’s multiple-comparison posttest (***P < 0.001, **P < 0.01, *P < 0.05)

Reciprocal hemizygosity analysis (RHA) and allele replacement analysis were, respectively, employed to validate the eight candidate causative genes in the QTLs (Table 2) based on the lethality and unavailability of their gene deletions. RHA was used for five non-essential genes including *RXT2*, *VID24*, *ECM22*, *IRA2* and *DAP1*, since their deletions were non-lethal. Allele replacement was used for two essential genes including *VPS34* and *AVO1*, whose null alleles are inviable, as well as the *CSC1* gene, whose deletion mutant was unavailable after several rounds of attempts. For RHA, five pairs of hemizygous diploid tp × sp hybrid strains were constructed (Additional file 1: Table S1), in which each pair retained a single copy of the superior (ScY01α-tp) or inferior (W65a-sp) parent allele of *RXT2*, *VID24*, *ECM22*, *IRA2* and *DAP1*, respectively, while the other copy of the gene was deleted. For allele replacement analysis, three pairs of allele homozygotes of diploid tp × sp hybrid strains were constructed (Additional file 1: Table S1), in which each pair contained two homogeneous gene allele from the superior (ScY01α-tp) or inferior parent (W65a-sp) allele of *VPS34*, *AVO1* and *CSC1*, respectively. The fermentation profiles of RHA and allele replacement strains at high temperature were shown in Additional file 1: Figure S1, and the diploid hybrid (tp × sp) of two parent strains was used as a control. To have better quantitative comparisons of fermentation capacities, the fermentation rates including maximum cell growth rate (*μ*_max_), glucose-consumption rate (*q*_s_max) and ethanol productivity (*P*_EtOH_) were calculated according to the fermentation data in Additional file 1: Figure S1, and shown in Fig. 5. From the results of RHA, compared with the control strain tp × sp, one of two hemizygotes for *VID24* and *DAP1* showed significantly decreased cell growth or/and fermentation capacities at high temperature (Fig. 5), whereas both two hemizygotes for *RXT2*, *ECM22* and *IRA2* showed increased thermotolerances to different extent (Fig. 5). As for allele replacement analysis, compared with the control strain tp × sp, one of two allele homozygotes for *VPS34* but both two allele homozygotes for *CSC1* and *AVO1* showed significantly increased cell growth or/and fermentation capacities at high temperature (Fig. 5). As for *VPS34* and *CSC1* in the major QTL2, the allele homozygotes containing the variants from the superior parent ScY01α-tp were expected to have higher thermotolerance than the control stain or the allele homozygotes containing the variants from the inferior parent W65a-sp. As for *AVO1* in the minor QTL3, the homozygote containing the variant from W65a-sp was expected to have higher thermotolerance than the control stain or the allele homozygote containing the variant from ScY01α-tp. Unexpectedly, the homozygote containing the *CSC1* allele from W65a-sp showed opposite results due to increased *P*_EtOH_ (Fig. 5). Overall, all these eight genes seemed to have impacts on high-temperature fermentation performance.

The detailed results were as follows: *VID24* was localized in the major QTL of QTL1 (Table 2). Deletion of the superior (ScY01α-tp) parent allele of *VID24* in the reciprocal hemizygote resulted in decreased cell growth at high temperature but not significantly, and had significant effects on *q*_s_max and *P*_EtOH_ at high-temperature (Fig. 5). This result suggested the *VID24* allele from the superior strain might act as a causative and positive gene in thermotolerance. *VPS34* was in the major QTL of QTL2 (Table 2). The allele homozygote containing two copies of the *VPS34*^D591E^ allele from the superior parent showed significantly higher fermentation rates and capacities at high temperature than the one containing two copies of the inferior parent allele as well as the control hybrid strain tp × sp (Fig. 5, Additional file 1: Figure S1). Furthermore, our previous genome sequencing showed that the diploid superior parent strain ScY01 has two homogenous copies of the *VPS34* ^D591E^ allele [30]. Therefore, the *VPS34*^D591E^ allele might be a causative gene in thermotolerance. *DAP1* was in the minor QTL of QTL4 (Table 2). The *DAP1*^V39I^ mutant allele inheriting from the inferior parent strain W65a-sp was found in the superior thermotolerant pool (Table 2, Additional file 5: Dataset S3). We estimated that the reciprocal hemizygote containing the inferior parent allele of *DAP1* might have higher thermotolerance than the one containing the superior parent allele of *DAP1*. Unexpectedly, the result is quite the opposite. Compared with the control strain tp × sp, the reciprocal hemizygote containing the inferior parent allele of *DAP1* showed significantly decreased fermentation rates and capacities at high temperature, while the one containing the superior parent allele of *DAP1* exhibited significantly increased thermotolerance (Fig. 5, Additional file 1: Figure S1). This result implicated that the inferior parent allele of *DAP1*^V39I^ might be a recessive deleterious mutation in segregants of the superior pool, while *DAP1* might act as a recessive beneficial gene in the superior thermotolerant parent. In terms of the other five genes except for *VID24*, *VPS34* and *DAP1*, the hybrid control strain tp × sp containing their heterogeneous alleles showed lower high-temperature fermentation performance than either the reciprocal hemizygotes only retaining a single copy of allele or the allele homozygotes containing two homogeneous copies of allele (Fig. 5, Additional file 1: Figure S1). The extensive loss of heterozygosity in *S. cerevisiae* genomes have been reported to enable the expression of recessive alleles and generating novel allele combinations with potential effects on phenotypic diversity [31]. Thus, loss of heterozygosity in the five gene alleles might play a similar function in contributing to high-temperature fermentation performance.

Overall, all the results suggested that these eight genes were probably causative genes that linked to high-temperature fermentation performance in *S. cerevisiae*, although in different ways and to different extent.

### Characterization of key causative gene alleles for improving high-temperature fermentation of the industrial yeast

**Fig. 6:**
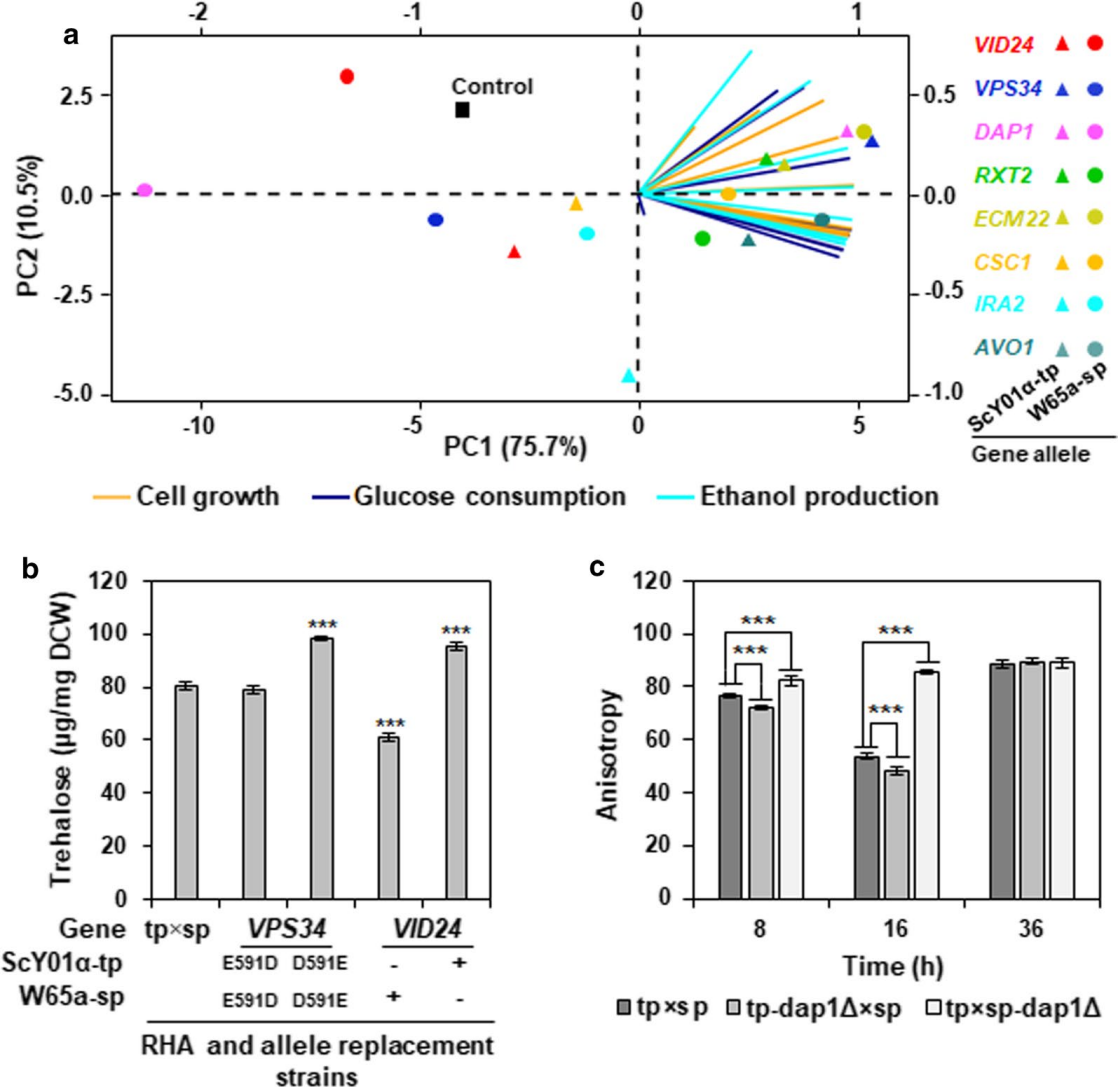
Principal component analysis of high-temperature fermentation data and physiological impacts of key causative genes. **a** Principal component analysis (PCA) of high-temperature fermentation data from all the RHA and allele replacement strains, including cell growth (orange lines), glucose consumption (purple lines) and ethanol production (turquoise lines) during fermentation (hours 0, 8, 12, 18, 24 30 36 42 and 48). Means of biological repeats (in duplicates) are used. The gene alleles originating from the superior (ScY01α-tp, triangle symbols) and inferior (W65a-sp, circle symbols) parents in the RHA and allele replacement strains were colour-coded. **b** Trehalose accumulation in the RHA and allele replacement strains of *VID24* and *VPS34* at high temperature. **c** Membrane fluidity of the RHA strains of *DAP1* at high temperature. The Membrane fluidity is determined by the steady-state anisotropy of fluorescent probe 1-[4-(trimethylamino)pheny]-6-phenyl-1,3,5-hexatriene (TMA-DPH). Yeast cells were grown at 42 °C in 100 ml Erlenmeyer flasks containing 50 ml YP medium with 200 g/l glucose at 220 rpm. For measuring trehalose accumulation, cells were harvested after incubation for 36 h. For determining membrane fluidity, cells were harvested after incubation for 8 h, 16 h and 36 h at the early-exponential, mid-exponential and stationary phases, respectively. Data represent the mean and standard error of duplicate cultures at each condition. Statistical analysis in **b** and **c** was performed using one-way ANOVA followed by Tukey’s multiple-comparison posttest (***P < 0.001, **P < 0.01, *P < 0.05)

To further distinguish good targets from the eight causative gene alleles for improving high-temperature fermentation of the industrial yeast, we performed principal component analysis (PCA) for high-temperature fermentation data from all the RHA and allele replacement strains at 42 °C in Additional file 1: Figure S1, including cell growth, glucose consumption and ethanol production at all the time points during fermentation. As shown in Fig. 6a, the first and second accounted for 75.7% (PC1) and 10.5% (PC2) of the total variation, respectively. The RHA and allele replacement strains harbouring gene allele of *VID24*, *VPS34* or *DAP1* from the superior industrial parent ScY01α-tp (red, blue and pink triangles in Fig. 6a), showing enhanced high-temperature fermentation capacities in contrast to the control stain tp × sp, were clearly separated by the PCs from the RHA and allele replacement strains containing those gene alleles from W65a-sp (red, blue and pink circles in Fig. 6a). This result confirmed that the alleles of *VID24*, *VPS34* and *DAP1* in the industrial yeast ScY01 could be greatly beneficial to high-temperature fermentation. By contrast, as for the rest five genes including *RXT2*, *ECM24*, *CSC1*, *IRA2* and *AVO1*, the RHA and allele replacement strains harbouring their alleles from the parents were relatively closely grouped by the PCs, although showing higher high-temperature fermentation capacities than the control strain tp × sp. This result suggested that the alleles of *RXT2*, *ECM24*, *CSC1*, *IRA2* and *AVO1* in the industrial yeast ScY01 might play minor roles in supporting high-temperature fermentation. Most strikingly, the PCs highly distinguished the RHA and allele replacement strains harbouring the superior gene alleles of *VPS34* or *DAP1* (Fig. 6a), suggesting that they would be good targets for improving high-temperature fermentation of the industrial yeast.

*VPS34* and *VID24* have been reported to be involved in the degradation of FBPase [32, 33], thus possibly affecting trehalose accumulation. Furthermore, trehalose is required on both sides of the lipid bilayer of membranes for effective protection against thermal stress in *S. cerevisiae* [34]. Thus, we measured the trehalose levels in cells of the RHA and allele replacement strains of *VPS34* and *VID24* and the control strain tp × sp, which were grown at thermal stress conditions (42 °C). Compared to the control strain tp × sp, the allele homozygote containing two copies of the *VPS34*^D591E^ allele from the superior parent had significantly higher trehalose levels (Fig. 6b), which was positively correlated with its enhanced high-temperature fermentation capacities (Fig. 5, Additional file 1: Figure S1). Similarly, the reciprocal hemizygote containing the superior (ScY01α-tp) parent allele of *VID24* showed significantly higher trehalose levels than the control strain, while the reciprocal hemizygote containing the inferior (W65a-sp) parent allele of *VID24* had significantly lower trehalose levels, positively correlating with their enhanced high-temperature fermentation capacities (Fig. 5, Additional file 1: Figure S1). These results indicated that the superior alleles of *VPS34* and *VID24* might achieve beneficial effects on the high-temperature fermentation capacities of the industrial yeast by increasing trehalose levels.

*DAP1* mutation leads to defects in sterol synthesis, and thus influencing membrane fluidity [35, 36]. Cell wall and membrane are the first defence barrier against environmental stresses. Negative correlation between stress tolerance and membrane fluidity has been observed for ethanol stress [37]. Therefore, we determined the membrane fluidity of the reciprocal hemizygotes of *DAP1* and the control strain tp × sp by measuring steady-state anisotropy of membrane-incorporated 1-[4-(trimethylamino)pheny]-6-phenyl-1,3,5-hexatriene (TMA-DPH). High anisotropy values indicate low membrane fluidity, allowing strong protection against environmental stresses, and vice versa. The reciprocal hemizygote containing the superior (ScY01α-tp) parent allele of *DAP1* exhibited enhanced high-temperature fermentation capacities (Fig. 5, Additional file 1: Figure S1). Positively correlated, this strain showed significantly higher anisotropy levels at the early-exponential (8 h), mid-exponential (16 h) phases than the control strain, indicating lower membrane fluidity (Fig. 6c), thus providing effective protection against thermal stress to support active cell metabolism, especially at the mid-log phase. By contrast, membrane fluidities of these cells at the stationary phase among the reciprocal hemizygotes of and the control strains. These results suggested that the superior allele of *DAP1* might achieve a beneficial effect on the high-temperature fermentation capacities of the industrial yeast by inhibiting membrane fluidity.

**Fig. 7:**
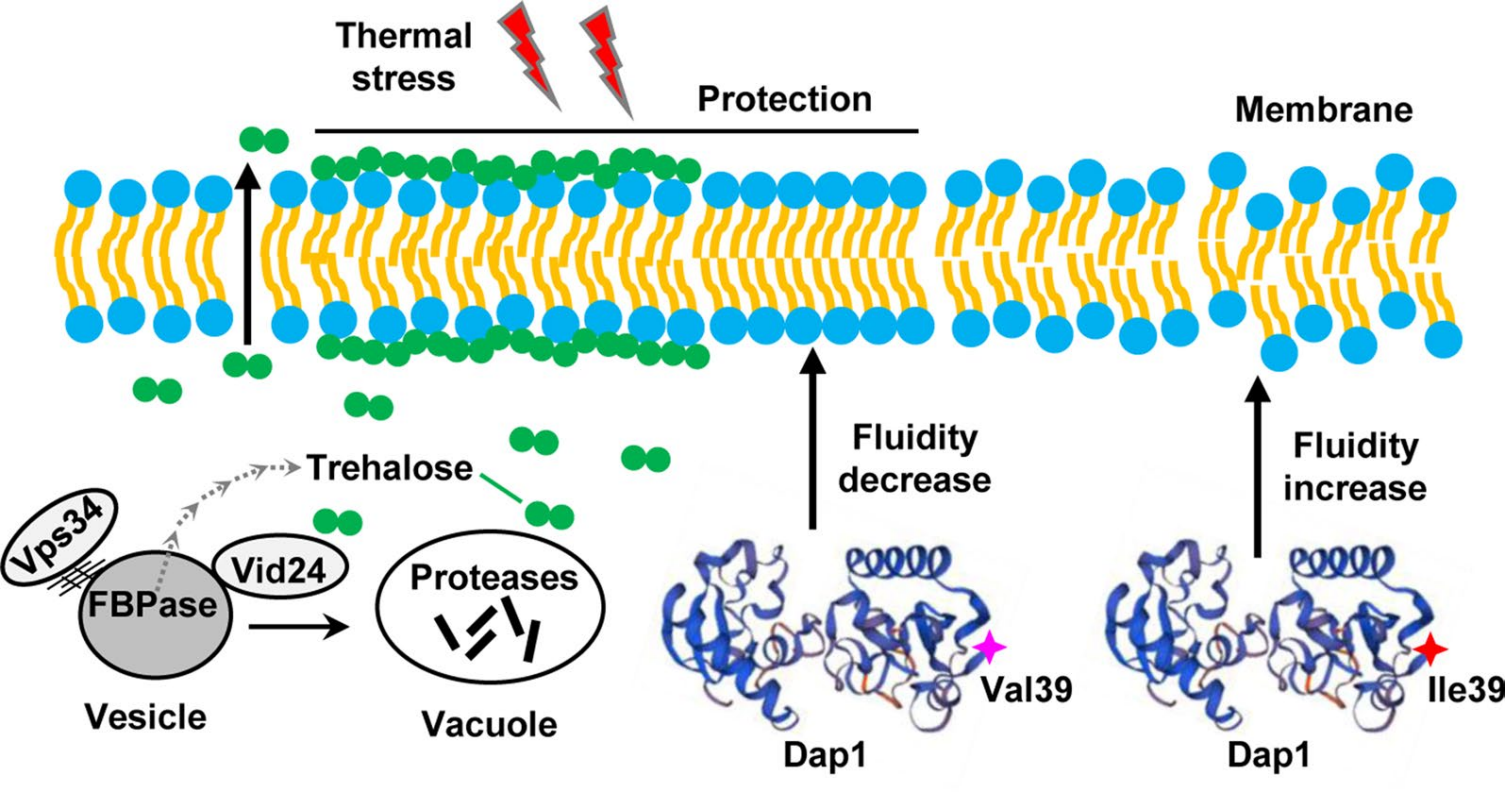
An overarching model integrating good targets for improving high-temperature fermentation of the industrial yeast

Based on characterization of key causative gene alleles, we generated an overarching model integrating good targets for improving high-temperature fermentation of the industrial yeast (Fig. 7). Remarkably, we found that the physiological beneficial effects of the superior (ScY01α-tp) parent alleles converged on cell membrane. Vps34 and Vid24 from the superior parent can contribute to trehalose accumulation at a high level, thus providing more trehalose on both sides of the lipid bilayer of membranes for effective protection against thermal stress. On the other hand, Dap1 containing Valine instead of Isoleucine at position 39 can contribute to reduce membrane fluidity, thus providing a strong defense barrier against thermal stress. Taken together, our model supported the previous understandings that trehalose accumulation and reduced membrane fluidity could promote high-temperature fermentation in industrial yeast [34, 37], meanwhile revealing *VPS34* and *DAP1* as good targets for further enhancing high-temperature fermentation of the industrial yeast.

## Discussion

Elevated thermotolerance is a highly valuable trait of industrial yeasts that can substantially reduce the production costs. Previous studies have identified several causative genes and gained some insights into the underlying mechanism of this complex trait via various efficient approaches, especially QTL methodology [5, 10, 12]. A major challenge of QTL analysis is to efficiently identify minor QTLs linked to the inferior parent strain. Since the phenotype is often masked by many subtle factors, for instance, epistasis [13], it is difficult to characterize the linkage between minor QTLs and the phenotype. However, minor QTLs are unignorable, because they may cause synergistic or additive effect, thus resulting in significant effects on the related phenotype as major QTLs. An efficient strategy has been used to reveal minor QTLs by eliminating candidate QTLs in both superior and inferior parent strains and repeatedly mapping the QTL with pooled-segregant whole-genome sequence analysis [5]. This approach was further upgraded to be carried out using relatively low numbers of segregants [20].

Based on the extensive pooled-segregant whole genome sequence analysis, we successfully identified two major QTLs (QTL1 and QTL2) and two minor QTLs (QTL3 and QTL4) localized on chromosome II, XII, XV, XVI, respectively (Fig. 4, Table 2). Similar to previous study [20], our work confirmed that relatively low numbers of segregants can be used for successful QTL mapping using pooled-segregant whole-genome sequence analysis. Besides two methods of EXPLoRA and MUTIPOOL used to detect QTLs, we also sequenced two individual segregants from the superior pool and used IGV to manually check SNP frequencies to facilitate more accurate detection of QTLs closely associated with thermotolerance. Four QTLs and eight nonsynonymous gene alleles were narrowed down from dozens of QTLs and hundreds of nonsynonymous SNP variants after QTL mapping, and finally validated to be causative factors related to yeast thermotolerance (Additional file 4: Dataset 2, Additional file 5: Dataset 3, Figs. 5, 6). Thus, the workflow used in this study could be feasible and effective for QTL mapping and identification of candidate causative genes.

Interestingly, among the eight validated causative genes, both *VID24* and *VPS34* were found to be involved in translocation and degradation of fructose-1,6-bisphosphatase (FBPase) in the vacuole. *VID24* encodes a peripheral protein on vacuole import and degradation (Vid) vesicles [38], which is required to transfer FBPase from the Vid vesicles to the vacuole for degradation [32]. *VPS34* encodes the sole phosphatidylinositol (Pl) 3-kinase in yeast, which is essential for autophagy [39], which is also required for the degradation of extracellular FBPase in the vacuole import and degradation (VID) pathway [33]. When yeast cells are out of glucose feeding for a long time, Vps34 is induced and co-localized with actin patches in starved cells. Once Vps34 is absent, FBPase and the Vid24 associated with related actin patches before and after re-feeding glucose. Strikingly, *VID24* null mutation leads to FBPase accumulation in the vesicles, thus affecting trehalose synthesis [32, 40]. *VPS34* null mutant also arrests FBPase with high levels in the extracellular fraction. A previous study indicated trehalose is beneficial to protect cells from thermal stress in *S. cerevisiae* [34]. Hence, we speculated that *VID24* and *VPS34* might affect trehalose synthesis by controlling the degradation of FBPase and thus be closely linked to thermotolerance. As expected, we observed the positive correlation between the accumulation of trehalose and the improvement of ethanol production due to the existent of *VID24* and *VPS34*^D591E^ originating from the thermotolerant parent strain ScY01α-tp (Fig. 6b). In terms of testing the relationship between the degradation of FBPase and the improvement of ethanol production, it would be worthwhile to be further investigated in the future.

*DAP1* was identified to be linked to thermotolerance by minor QTL mapping (Fig. 4b). *DAP1* encodes Heme-binding protein and mutations lead to defects in mitochondria, telomeres, and sterol synthesis [35, 36], which was closely associated with thermotolerance [10, 12]. The abundance and composition of sterol plays a significant modulatory role in yeast response to thermal stress by affecting membrane fluidity [41]. Furthermore, the reciprocal hemizygote containing the superior allele of *DAP1* showed increased high-temperature fermentation and lower membrane fluidity in contrast to the control strain (Fig. 6c). Thus, *DAP1* might be involved in thermotolerance by affecting sterol synthesis and membrane fluidity. Furthermore, our results suggested *DAP1* to be a recessive causative gene linked to thermotolerance, which was influenced by the genetic background. The mutant allele of *DAP1*^V39I^ from the inferior parent was validated to be a recessive deleterious mutation for thermotolerance, since the hemizygote containing the *DAP1*^V39I^ allele showed decreased high-temperature fermentation performance compared to the hybrid control strain tp × sp (Fig. 5c). Meanwhile, the wild-type *DAP1* allele was validated to be a recessive beneficial gene in the superior parent, since the hemizygote containing the wild-type *DAP1* allele showed increased high-temperature fermentation performance compared to the hybrid control strain tp × sp (Fig. 5c). A previous study reported that mechanisms of hydrolysate tolerance are very dependent on the genetic background, and causal genes in different strains are distinct [24]. Our results confirmed that the effect of recessive alleles or variants might be covered by different genetic backgrounds and complementation of recessive alleles could also contribute to the strain improvement.

Recent genome-wide association study revealed an extensive loss of heterozygosity (LOH) associated with phenotypic diversity across 1011 *S. cerevisiae* isolates [31]. LOH could provide a driving force of evolution during the adaptation of the hybrid strain to novel or stressful environments by enabling the expression of recessive alleles to potentially support the robustness of cells [42, 43]. In this study, based on RHA and allele replacement analysis, positive effects of LOH on high-temperature fermentation were observed for five causative genes including *RXT2*, *ECM24*, *CSC1*, *IRA2* and *AVO1* identified by QTL mapping (Figs. 5, 6). Furthermore, we found that the heterozygous forms of these five genes in the control strain tp × sp seemed to have negative effects on thermotolerance. This was different from the findings that the beneficial mutations in heterozygous form seemingly confer no benefit at the cellular level in nystatin [44]. These results suggested that LOH would be an interesting focus for QTL analysis studies.

## Conclusions

We evaluated high-temperature fermentation performances of 124 industrial, natural or laboratory *S. cerevisiae* strain and selected one superior thermotolerant strain and one inferior thermosensitive strain as parent strains. Pooled-segregant whole-genome sequence analysis was performed for the selected three segregant pools including the superior, inferior and random pools from the hybrid of those two parent strains. Two individual segregants in the superior pool were also sequenced to facilitate the detection of nonsynonymous variants linked to thermotolerance. Candidate causative genes were validated by RHA and allele replacement. Finally, two major QTLs and two minor QTLs as well as eight causative genes containing nonsynonymous SNP variants were identified to be closely linked to yeast thermotolerance. Strikingly, the superior parent alleles of *VPS34*, *VID24* and *DAP1* converged on cell membrane by increasing trehalose accumulation or reducing membrane fluidity, and thus beneficial to high-temperature fermentation of the industrial yeast. Furthermore, LOH of five causative genes including *RXT2*, *ECM24*, *CSC1*, *IRA2* and *AVO1* had positive effects on high-temperature fermentation, suggesting that LOH would be an interesting focus for QTL analysis studies. Overall, we identified novel causative genes linked to high-temperature fermentation performance of yeast, providing guidelines to develop more robust thermotolerant strain for the industrial production of ethanol.

## Methods

### Strains, cultivation conditions and sporulation

All the strains used in this study are listed in Additional file 1: Table S1. Yeast cells were grown in YPD media (per litre, 10 g yeast extract, 20 g peptone, 20 g glucose) or on YPD agar plates supplemented with 20 g/l agar. Gene knockout transformants or segregants were selected on YPD agar plates containing 400 µg/ml geneticin, 70 μg/ml zeocin or 200 μg/ml hygromycin B as specified in the text. Mating, sporulation and isolation of haploid segregants were conducted by following standard procedures [45]. The *MATα* and *MATa* haploid segregants of parent strains were isolated from strains ScY01 and W65, and named ScY01α and W65a, respectively. To avoid mating-type switch [46], the *HO* gene in ScY01α and W65a were further knocked out using the previously reported method based on PCR amplification and one-step gene replacement [47]. Zeocin- and geneticin-resistance cassettes were PCR amplified from the plasmids pREMI-Z [48] and pFA6-kanMX4 [49] (Additional file 1: Table S1), flanked with 500-bp homologous sequences to the *HO* gene by fusion PCR and transferred into ScY01α and W65a using the electrotransformation method [50], respectively. Positive transformants were separately selected on zeocin and geneticin selective plates. To confirm successful knockout, diagnostic PCR reactions with primers designed on the HO locus as well as zeocin- and geneticin-resistance cassettes were used (for primers, see Additional file 6: Table S3). The resulting haploid parent strains were named ScY01α-tp and W65a-sp, respectively, and then crossed and sporulated. Since zeocin- and geneticin-resistance cassettes were allelic in the hybrid diploid strain tp × sp, successfully segregated haploid spores should only inherit one drug resistance capacity of either zeocin or geneticin. Thus, to select haploid segregants, sporulated cells were first isolated on YPD agar plates, and then replica plated on both zeocin and geneticin selective plates. Cell patches only grown on zeocin or geneticin selective plate were further subjected to diagnostic PCR for the MAT locus to determine the mating type of segregants and confirm haploidy [51].

### Thermotolerance phenotyping

The thermotolerance phenotypes of yeast cells were determined by three evaluation ways as specified in the text: (1) cell growth at 42 °C monitored by measuring the OD_600_ at the 24-h time point, (2) stress tolerance index (STI) based on cell growth (calculated as the ratio of the OD_600_ at 42 °C versus the OD_600_ at 30 °C measured at the 16-h time point), and (3) fermentation capacity at 42 °C by measuring cell growth, glucose consumption and ethanol production. Cell growth experiments were performed either using high-throughput growth assays in 96-well plates containing 1 ml YPD medium or using 50-ml Falcon tubes containing 10 ml YPD medium with shaking at 220 rpm. Fermentation experiments were conducted in 100-ml Erlenmeyer flasks containing 50 ml YP medium (per liter, 10 g yeast extract, 20 g peptone) with 200 g/l glucose at 220 rpm. Cells were pre-cultured in YPD medium at 30 °C overnight before applying to cell growth or fermentation experiments. Starting OD_600_ used in all the experiments was 0.2. Optical density (OD) at 600 nm was measured using a platereader (Molecular Devices SpectraMax M2e, San Jose, CA, USA). Concentrations of glucose and ethanol were monitored by high-performance liquid chromatography (HPLC) on an Agilent 1260 system (Agilent, Santa Clara, CA, USA) equipped with a refractive index detector and a Phenomenex RFQ fast acid column (100 mm × 7.8 mm ID) (Phenomenex Inc., Torrance, CA, USA). The column was eluted with 0.01 N H_2_SO_4_ at a flow rate of 0.6 ml min^−1^ at 55 °C.

### Pooled-segregant whole-genome sequence analysis

After crossing the two parent haploid strains ScY01α-tp and W65a-sp, the ten most thermotolerant segregants were assembled in the superior pool, the ten most thermosensitive segregants were assembled in the inferior pool and ten random segregants were used to assemble the random pool. The segregants were grown separately in 50 ml liquid YPD media at 30 °C to exponential phase. Each pool was made by mixing equal amounts of cells from the ten segregants based on OD_600_ as previously described [52] and subjected to whole-genome resequencing. Besides, the haploid parent strains ScY01α-tp and W65a-sp and two individual segregants including the best and worst spores (G28 and Z118) in the superior pool were subjected to whole-genome resequencing as well. Genomic DNA isolation and the sequencing libraries were constructed and sequenced on Illumina HiSeq 4000 using 150-bp paired-end sequencing by the Beijing Genomics Institute (BGI) (Shenzhen, China). A mean of 15.9 million 150-bp clean reads was generated for each library. All the genome sequencing raw data were deposited in the Sequence Read Archive (SRA) at the National Center for Biotechnology Information (NCBI) under the BioProject ID PRJNA414133 with accession number SRP119879.

### Variant detection, QTL mapping and identification of candidate causative genes

Variants were detected using the Genome Analysis Toolkit (GATK v3.5) Best Practices pipeline [53, 54]. The *S. cerevisiae* S288c genome was used as a reference and downloaded from RefSeq at the NCBI (sequence assembly version R64, RefSeq assembly accession: GCF_000146045.2). Initially called SNPs were filtered with a minimum read depth of 20 or a minimum variant frequency of 80%. SNP frequency was initially defined by using the percentage of SNP-containing reads in total mapped reads spanning each locus as previously reported [55]. Variant annotation was performed using the package ANNOVAR [56], and variants were then called using GATK HaplotypeCaller to generate variant lists of sequenced samples, relative to the S288c reference genome. By comparing the variant lists of two parent strains ScY01α-tp and W65a-sp, total 35,459 segregating discordant SNP sites were used as genetic makers for QTL analysis (Additional file 3: Dataset S1).

We used the EXPLoRA method [26], to identify large chromosomal regions containing QTLs, and subsequently analysed those regions with the MULTIPOOL method [57] to obtain high-resolution predictions for causative QTL regions. First, variants of the superior pool were analysed by EXPLoRA (version 1.0) to identify all the putative QTLs when the posterior probability assigned to the marker is larger than 0.95 (Additional file 4: Dataset S2). Second, for each QTL-containing region identified with the EXPLoRA method, SNP allele frequencies in superior pool versus inferior pool and superior pool versus random pool were compared using MULTIPOOL (-n 1000, -c 3300, -r 100 –m contrast) to generate the high-resolution QTL map (Additional file 4: Dataset S2). When one locus showed a peak of allele frequencies with the EXPloRA method and simultaneously had a maximum LOD (log10 likelihood ratio) value higher than 5 in superior pool versus inferior pool and random pool with the MULTIPOOL method, this locus was identified as a candidate causative QTL. Additionally, since the inferior parent with low thermotolerance might contain recessive beneficial variants to thermotolerance, putative QTLs linked with the inferior parent were also analysed using the genome variants of the inferior parent as a reference. QTLs linked to the superior and inferior parents were named major and minor QTLs, respectively.

Variants in QTLs resulting in nonsynonymous mutation were annotated using the package ANNOVAR [56] (Additional file 5: Dataset S3). To further narrow down and identify candidate causative variants and their affected genes, we first sorted out the variants, which in two sequenced individual spores G28 and Z118 were similar to those in the parent strains and also consistent with those in the superior pool. Second, we manually checked the variant frequencies of these variants in the sequenced segregant pools using Integrative Genomics Viewer (IGV) [27–29]. Only the variants in major QTLs meeting the criteria of allele frequencies with ≤ 10% in the inferior pool, ≥ 75% in the superior pool and around 50% in the random pool as well as the variants in minor QTLs meeting the criteria of allele frequencies with ≤ 25% in the inferior pool, ≥ 75% in the superior pool and around 50% in the random pool were considered to be causative variants related to thermotolerance (Additional file 5: Dataset S3).

### Reciprocal hemizygosity analysis (RHA) and allele replacement

To validate the causative genes within QTLs, RHA was used for non-essential genes including *RXT2*, *VID24*, *ECM22*, *IRA2* and *DAP1*, whereas allele replacement was used for essential genes including *VPS34* and *AVO1* whose null alleles are inviable. Additionally, since *CSC1* deletion mutant for RHA failed to be obtained after several rounds of attempts, allele replacement was also used for this non-essential gene. RHA was carried out as described previously [17]. PCR-mediated gene disruption, based on homologous recombination, was used to generate gene null mutants [58]. The gene disruption cassettes containing hygromycin modules flanked by 500-bp homologous sequences to the target genes were obtained using fusion PCR. Hygromycin-resistance modules were PCR amplified from the plasmid pRS426-hphB [59] (Additional file 1: Table S1). The 500-bp homologous sequences upstream and downstream the five non-essential genes were PCR amplified from ScY01-tp genomic DNA. The primers were supplemented in Additional file 6: Table S3. The gene disruption cassettes were transferred into ScY01α-tp and W65a-sp using the electrotransformation method [50], respectively. Positive transformants were selected on hygromycin selective plates. Successful gene disruptions were confirmed by diagnostic PCR reactions with primers designed on the target genes as well as hygromycin-resistance cassette (for primers, see Additional file 6: Table S3). Subsequently, for each non-essential causative gene candidate, the gene disruption mutant of ScY01α-tp and the wild-type strain of W65a-sp or vice versa were crossed to construct the diploid hybrid, which was the reciprocal hemizygote that only contained one single gene allele from either ScY01α-tp or W65a-sp.

Allele replacement was achieved using PCR-based fragment through homologous recombination. The 5′ homologous sequence contained the region from nearly 500 bp upstream the identified SNP by QTL mapping to the stop codon in the target gene. The 3′ homologous sequence contained the region 500 bp downstream the stop codon of the target gene. The hygromycin-resistance module was PCR fused between the 5′ and 3′ homologous sequences. The homologous recombination fragment containing the identified SNP by QTL mapping in the target gene from one parent was transformed into the other parent or vice versa. Positive colonies were screened on hygromycin selective plates and subjected to PCR amplification and Sanger sequencing to confirm allele replacement (for primers, see Additional file 6: Table S3). Subsequently, the allele replacement mutant of ScY01α-tp and the wild-type strain of W65a-sp or vice versa were crossed to construct the diploid hybrid, which was the allele homozygote that contained two homogeneous gene allele from either ScY01α-tp or W65a-sp. Fermentation capacities of all the reciprocal hemizygotes and allele homozygotes were evaluated using the hybrid diploid tp/sp as a control.

### Determination of trehalose and membrane fluidity

Yeast cells were grown at 42 °C in 100-ml Erlenmeyer flasks containing 50 ml YP medium with 200 g/l glucose at 220 rpm. For measuring trehalose accumulation, cells were harvested at stationary phase after incubation for 36 h, when cells accumulate high levels of trehalose as previously reported [60]. Trehalose levels were determined using trehalose content detection kit (BestBio, China) in accordance with the manufacturer’s instructions. For determining membrane fluidity, cells were harvested after incubation for 8 h, 16 h and 36 h at the early-exponential, mid-exponential and stationary phases, respectively. Membrane fluidity was assessed using steady-state fluorescence spectroscopy. Steady-state anisotropy of 1-[4-(trimethylamino)pheny]-6-phenyl-1,3,5-hexatriene (TMA-DPH, MedChemExpress, USA) following incorporation of the probe into yeast plasma membranes was measured, as previously described with a slight modification [61]. A Spark™ Multimode Microplate Reader (Spark 10 M, Tecan, Switzerland) was used for the measurement of the steady-state anisotropy of TMA-DPH. Both labelling of cells with TMA-DPH and the measurement were conducted at 42 °C.

### Calculation of fermentation rates, statistical significance tests and principal component analysis

Fermentation parameters including maximum cell growth rate (*μ*_max_), maximum glucose consumption rate (*q*_s_max) and ethanol productivity (*P*_EtOH_) were calculated corresponding to the fermentation profiles using Originlab^®^ Origin 8 as previously reported [62]. For comparison of high-temperature fermentation between the control strain tp × sp and RHA or allele replacement strains, one-way ANOVA was used, followed by Tukey’s multiple-comparison posttest with a 95% confidence interval. Statistics were performed using Origin (version 8.0). The differences were considered significant at three levels of *P* < 0.001, *P* < 0.01 and *P* < 0.05. Principal component analysis (PCA) was used to evaluate the impact of gene alleles in the RHA and allele replacement strains, respectively, originating from the superior and the inferior parent, on cell growth, glucose consumption and ethanol production at high temperature (42 °C) during fermentation (hours 0, 8, 12, 18, 24 30 36 42 and 48). Packages FactoMineR and Factoextra [63] were used within R environment [64] for the PCA data analysis and ggplot2-based visualization, separately.

## Additional files

**Additional file 1: Table S1.** *S. cerevisiae* strains used in this study. **Figure S1.** Fermentation profiles of RHA and allele replacement strains of the causative genes.

**Additional file 2: Table S2.** All the data in Fig. 2a.

**Additional file 3: Dataset S1.** Markers for QTL analysis in two parent strains.

**Additional file 4: Dataset S2.** QTL lists detected by the EXPLoRA and MULTIPOOL methods.

**Additional file 5: Dataset S3.** Annotation of SNP variants in major and minor QTLs detected by MULTIPOOL.

**Additional file 6: Table S3.** Primers used in this study.

## Abbreviations

HTF: high-temperature fermentation
QTL: quantitative trait loci
RHA: reciprocal hemizygosity analysis
STI: stress tolerance index
SNPs: single nucleotide polymorphisms
IGV: Integrative Genomics Viewer
FBPase: fructose-1,6-bisphosphatase
Pl: phosphatidylinositol
VID: vacuole import and degradation
LOH: loss of heterozygosity
HPLC: high performance liquid chromatography
BGI: Beijing Genomics Institute
SRA: Sequence Read Archive
PCA: principal component analysis
TMA-DPH: 1-[4-(trimethylamino)pheny]-6-phenyl-1,3,5-hexatriene
