# Correction to: QTL analysis reveals genomic variants linked to high-temperature fermentation performance in the industrial yeast

**DOI:** 10.1186/s13068-019-1425-8

**Published:** 2019-04-10

**Authors:** Zhen Wang, Qi Qi, Yuping Lin, Yufeng Guo, Yanfang Liu, Qinhong Wang

**Affiliations:** 1 CAS Key Laboratory of Systems Microbial Biotechnology, Tianjin Institute of Industrial Biotechnology, Chinese Academy of Sciences, Tianjin, 300308 China; 2 University of Chinese Academy of Sciences, Beijing, 100049 China

## Correction to: Biotechnol Biofuels (2019) 12:59 10.1186/s13068-019-1398-7

Shortly after publication of the original article [1], the authors noticed a few errors which are outlined below:

In Table 2, the SNP mutation for *RXT2* in ScY01α-tp should be 331 C>G. As shown in Additional file 5: Dataset S3, the position of the SNP in *RXT2* is NC_001134.8:435,368, and annotated as G>C according to the chromosome sequence. Since the coding sequence of *RXT2* (systematic gene name: YBR095C) is on the Crick strand, the SNP should be described as C>G at the 331 position according to the coding sequence. The corrected Table 2 is provided here.

**Table 2:** Genes with nonsynonymous variants in two major and two minor QTLs

QTLs	Chr	Start (bp)	End (bp)	Length (bp)	LOD score	Affected gene	Mutation (S288c genome as a reference)	
							ScY01α-tp	W65a-sp
Major QTLs								
QTL1	II	408,800	553,700	144,900	17	*RXT2*	331 C>G (R111G)	Wild type
						*VID24*	154 C>T (P52S)	Wild type
QTL2	XII	595,800	633,500	37,700	300	*ECM22*	1954 G>A (G652S)	Wild type
						*VPS34*	1773 C>G (D591E)	Wild type
						*CSC1*	1126 C>A (Q376K)	Wild type
Minor QTLs								
QTL3	XV	174,500	184,900	10,400	272	*IRA2*	Wild type	7222 C>A (P2408T)
						*AVO1*	Wild type	2558 T>C (V853A)
QTL4	XVI	228,200	238,100	9900	155	*DAP1*	Wild type	115 G>A (V39I)

The gene *ECM24*, which was misspelled six times in the original article, should be *ECM22* in the following contents: the Abstract, the last paragraph of the Background, sentence 5 and 6 in paragraph 1 under the heading “Characterization of key causative gene alleles for improving high-temperature fermentation of the industrial yeast”, the last paragraph of the Discussion and the Conclusions.

Page 11, paragraph 3 under the heading “Characterization of key causative gene alleles for improving high-temperature fermentation of the industrial yeast”, sentence 8 should be: “By contrast, membrane fluidities of these cells at the stationary phase among the reciprocal hemizygotes of *DAP1* and the control strains were similar.”

In the following two contexts, the word “thermosensitive” should be “thermotolerant”. Page 4, paragraph 2 under the heading “Screening of the superior, inferior and random pools of segregants for genome sequencing”, sentence 7 should be: “Thus, ten segregants showing the 10 highest OD_600_ ratios (1.37 to 2.17) than ScY01α-tp were selected as the most thermotolerant segregants and assembled in the superior pool.” Sentence 3 in the Fig. 3b legend should be: “Ten segregants showing the 10 highest OD_600_ ratios (1.37 to 2.17) than ScY01α-tp were selected as the most thermotolerant segregants and assembled in the superior pool.”

In sentence 2 of the Fig. 4 legend, the strain name “W65a-tp” should be “W65a-sp”. In sentence 4 of the Fig. 4 legend, the Ref. [30] should be [25].

Page 14, Methods paragraph 2, sentence 5 should be: “Starting OD_600_ used in all the experiments was 0.5.”

The description “(hours 0, 8, 12, 18, 24 30 36 42 and 48)” should be “(hours 0, 4, 8, 12, 18, 24, 30, 36, 42 and 48)” in the following two contents: sentence 1 in the Fig. 6 legend and sentence 5 in the last paragraph of the Methods.

These changes will in no manner affect the outcome/interpretation of the experiments as described in the original publication. The authors apologize for any inconvenience caused.
