# Supplementary material for: QTL analysis reveals genomic variants linked to high-temperature fermentation performance in the industrial yeast
Source: Biotechnol Biofuels. 2019 Mar 19;12:59. doi: 10.1186/s13068-019-1398-7 (PMC6423876; doi:10.1186/s13068-019-1398-7)
Supplement: Supplementary file 1 — Additional file 1: Table S1. S. cerevisiae strains used in this study. Figure S1. Fermentation profiles of RHA and allele replacement strains of the causative genes. [file 13068_2019_1398_MOESM1_ESM.pdf]

Table S1. *S. cerevisiae* strains used in this study

Strain	Description	Reference or source
W1 to W121	*S. cerevisiae* strain collection from industry, lab and nature	Our lab collection
ScY01	Diploid strain, evolved thermotolerant strain derived from Ethanol Red	[11]
W65	Diploid strain, thermosensitive strain	Our lab collection
ScY01α	Haploid segregant from ScY01 with similar thermotolerance, *MATα*	This study
W65a	Haploid segregant from W65 with similar thermosensitivity, *MATa*	This study
ScY01α-tp	Haploid segregant from ScY01 with similar thermotolerance, *MATα, ho*::BleoR	This study
W65a-sp	Haploid segregant from W65 with similar thermosensitivity, *MATa, ho*::KanMX4	This study
tp×sp	Hybrid diploid strain obtained by crossing ScY01α-tp and W65a-sp	This study
tp-*rxt2*Δ×sp	Hybrid diploid strain, ScY01α-tp (*rxt2::*hphB) crossed with W65a-sp	This study
tp×sp-*rxt2*Δ	Hybrid diploid strain, ScY01α-tp crossed with W65a-sp (*rxt2::*hphB)	This study
tp-*vid24*Δ×sp	Hybrid diploid strain, ScY01α-tp (*vid24::*hphB) crossed with W65a-sp	This study
tp×sp-*vid24*Δ	Hybrid diploid strain, ScY01α-tp crossed with W65a-sp *vid24:*hphB)	This study
tp-*ecm22*Δ×sp	Hybrid diploid strain, ScY01α-tp (*ecm22::*hphB) crossed with W65a-sp	This study
tp×sp-*ecm22*Δ	Hybrid diploid strain, ScY01α-tp crossed with W65a-sp (*ecm22::*hphB)	This study
tp-*vps34*^{E591D}×sp	Hybrid diploid strain, ScY01α-tp containing the *vps34*^{E591D} allele from W65a-sp crossed with W65a-sp	
tp×sp-*vps34*^{D591E}	Hybrid diploid strain, ScY01α-tp crossed with W65a-sp containing the *vps34*^{D591E} allele from ScY01α-tp	This study
tp-*csc1*^{K376Q}×sp	Hybrid diploid strain, ScY01α-tp containing the *csc1*^{K376Q} allele from W65a-sp crossed with sp	This study
tp×sp-*csc1*^{Q376K}	Hybrid diploid strain, ScY01α-tp crossed with W65a-sp containing the *csc1*^{Q376K} allele from ScY01α-tp	This study
tp-*ira2*Δ×sp	Hybrid diploid strain, ScY01α-tp (*ira2::*hphB) crossed with W65a-sp	This study
tp×sp-*ira2*Δ	Hybrid diploid strain, ScY01α-tp crossed with W65a-sp (*ira2::*hphB)	This study
tp-*avo1*^{V853A}×sp	Hybrid diploid strain, ScY01α-tp containing the *avo1*^{V853A} allele from W65a-sp crossed with W65a-sp	This study
tp×sp-*avo1*^{A853V}	Hybrid diploid strain, ScY01α-tp crossed with W65a-sp containing the *avo1*^{A853V} allele from ScY01α-tp	This study
tp-*dap1*Δ×sp	Hybrid diploid strain, ScY01α-tp (*dap1::*hphB) crossed with W65a-sp	This study
tp×sp-*dap1*Δ	Hybrid diploid strain, ScY01α-tp crossed with W65a-sp (*dap1::*hphB)	This study
**Plasmids**		
pREMI-Z	Zeocin-resistance plasmid containing zeocin resistance cassette (BleoR)	[46]
pFA6-kanMX4	Geneticin-resistance plasmid containing KanMX4 module	[47]
pRS426-hphB	Hygromycin-resistance plasmid by replacing *URA3* coding region with the hygromycin B resistance gene from *Escherichia coli*	In our lab, [56]

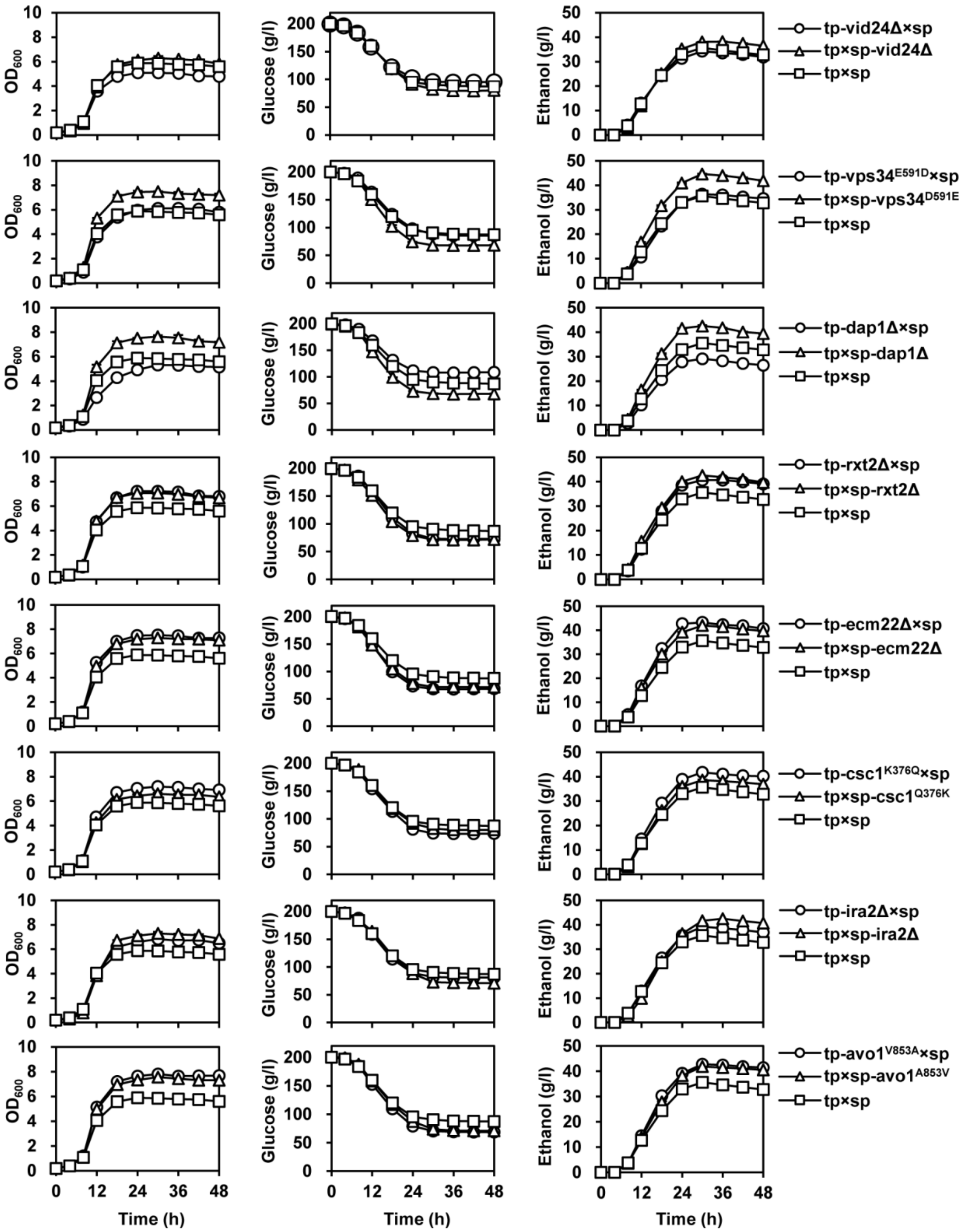

Figure S1. Fermentation profiles of RHA and allele replacement strains of the causative genes.

The RHA and allele replacement strains were detailed in Additional file 1: Table S1. High-temperature fermentation capacities were evaluated at 42°C in 100-

ml Erlenmeyer flasks containing 50 ml YP medium with 200 g/l glucose at 220 rpm. Data represent the mean and standard error of duplicate cultures at each condition (some error bars are covered by symbols).
